# Editorial: Telomere length and species lifespan

**DOI:** 10.3389/fgene.2023.1199667

**Published:** 2023-04-17

**Authors:** Kurt Whittemore, Michael Fossel

**Affiliations:** ^1^ Harvard Medical School, Boston, MA, United States; ^2^ Telocyte, Grand Rapids, MI, United States

**Keywords:** telomeres, species lifespan, body mass, telomere shortening rate, cellular senescence

The extent to which telomeres, the protective caps at the ends of chromosomes, determine lifespan and impact the aging process has been debated for many decades and continues to be debated today. In the late 1800s, the German evolutionary biologist August Weismann was one of the first to propose that aging is caused by the inability of cells to replicate forever (Strehler, 2000). His theory was seemingly disproved when Alexis Carrel published papers claiming that mammalian cells have an infinite capacity for replication (Carrel, 1912). This was the prevailing view for many decades until 1961 when Leonard Hayflick was able to use better techniques to demonstrate that mammalian cells do indeed have a finite replicative capacity (Hayflick and Moorhead, 1961). Through a series of discoveries (Watson, 1972; Szostak and Blackburn, 1982; Greider and Blackburn, 1985; Cooke and Smith, 1986; Greider and Blackburn, 1989), scientists soon worked out that the telomere caps at the ends of chromosomes shortened with each cell division, that cells entered into a state of senescence once telomeres became critically short, and that the enzyme telomerase could lengthen telomeres. There seemed to be a strong link between telomeres and aging. This relationship became more complicated however, when the discovery was made that mice lacking the gene for telomerase did not show dramatically shortened lifespans or defects until the third or fourth generation of breeding after the gene knockout (Blasco et al., 1997; Lee et al., 1998; Rudolph et al., 1999). Additionally, some species of mice have short telomere lengths similar to humans, while others have extremely long telomere lengths, yet they have approximately the same lifespans (Hemann and Greider, 2000). Today there are those who argue that other aspects of biology such as changes of DNA methylation with age are much more important determinants of the aging process. One approach to disentangle the effects of telomeres on the aging process is to study how telomeres affect the lifespan of different species. One recent article has demonstrated that the telomere shortening rate can be used to predict the lifespan of a variety of species (Whittemore et al., 2019), but there are still many additional questions that can be addressed and species that could be investigated.

In this small Research Topic of articles, the effect of telomeres in different species is explored. One of the articles found that there is little correlation between telomere length and lifespan in marine mammals (https://www.frontiersin.org/articles/10.3389/fgene.2021.737860/full). However, there was a strong correlation between body size and lifespan. These findings are in agreement with a previous study which found no correlation between initial species telomere length and lifespan but did find a strong correlation between telomere shortening rate and lifespan (Whittemore et al., 2019). In the marine mammal study, telomere shortening rates could not be determined since the necessary samples were not available. Another article in this Research Topic studied the telomere length of Psittacidae species (parrots) and found that longer living species had longer telomere lengths and greater antioxidant capacity (https://www.frontiersin.org/articles/10.3389/fgene.2023.1156730/full). Interestingly, the study found that breeding shortened telomere length. Another study investigated the paternal effects of telomere length on *Passer domesticus* (house sparrows) and found that older fathers had daughters with longer telomere lengths (https://www.frontiersin.org/articles/10.3389/fgene.2022.880455/full). A separate study in this Research Topic investigated the effects of the tumor suppressor genes p16 and p21 in a mouse model of Werner syndrome lacking telomerase (https://www.frontiersin.org/articles/10.3389/fgene.2021.597566/full). The study found that p16 and p21 had very different effects: p21 deficiency resulted in a dramatic increase in DNA damage responses, cellular senescence, apoptosis, and proliferation, whereas p16 deficiency showed reduced cellular senescence, apoptosis, increased telomere length, and increased cellular proliferation, ultimately rescuing the aging Werner syndrome phenotype.

The overall trend that species lifespan increases with increasing body mass, as also noted in the marine mammal article in this Research Topic (https://www.frontiersin.org/articles/10.3389/fgene.2021.737860/full), is quite fascinating. One possible explanation for this trend is simply that smaller animals are more likely to be eaten, and therefore, there would be no natural selection pressure to select for genes to allow for long life since the average lifespan would already be short. However, smaller organisms also often have higher heartbeat rates and metabolisms. Why is it that small animals have higher heartbeat rates and metabolisms? One proposal is that a faster metabolism is necessary in small organisms due to their large surface area to volume ratio through which heat can be lost (Levine, 1997). Thus, it is necessary for these species to have a fast metabolism in order to maintain their body temperature. Perhaps the fast metabolism of these species leads to more rapid cell turnover and telomere shortening. Note that within a given species, the opposite trend is observed: instead of large body size being correlated with longer life, smaller breeds within a species tend to live longer than larger breeds. For example, smaller breeds of dogs live longer on average than larger breeds of dogs (Selman et al., 2013), smaller breeds of horses live longer than larger breeds of horses (https://equestrianspace.com/average-lifespan-of-a-horse/), smaller mice live longer (Miller et al., 2000), and there is even a trend for shorter humans to live longer than taller humans (Samaras et al., 2003).

There are many important questions remaining to address telomere length and species lifespans. For example, how can one explain the large variation in mouse species telomere lengths, and the lack of large variation in the lifespan of those species? For example, the *Mus musculus castaneus* mouse species has a telomere length of 18–20 kb (Hemann and Greider, 2000), and yet the lifespan of *Mus musculus castaneous* (≈681 days (Hemann and Greider, 2000)) is similar to the lifespan of other mouse species such as C57BL/6J (≈767 days (Hemann and Greider, 2000)) which can have telomere lengths of 40–50 kb (Zijlmans et al., 1997; Hemann and Greider, 2000; Vera et al., 2012; Varela et al., 2016). A thorough investigation comparing initial telomere length, telomere shortening rate, percentage of short telomeres, the length of the shortest telomeres, and other aging markers such as DNA methylation may provide insights. Another caveat is that telomeres are often measured from circulating lymphocytes in the blood, which may not be the best cell type for these measurements (Fossel, 2012). Additional Research Topics of interest are more thorough investigations of outlier species which have much longer lifespans than expected such as the naked mole rat which can live 31 years (Buffenstein, 2005) compared to the mouse which lives about 2 years (Hemann and Greider, 2000), or the bat, which can live up to 37 years in some species (de Magalhães et al., 2007) even though a bat is about the same size as a mouse. Overall, a complete understanding of telomere dynamics and species lifespan requires further studies and discoveries.
